# Rural-Urban Differences in Breast Cancer Stage at Diagnosis

**DOI:** 10.1089/whr.2021.0082

**Published:** 2022-02-14

**Authors:** Gabrielle LeBlanc, Inkoo Lee, Henry Carretta, Yi Luo, Debajyoti Sinha, George Rust

**Affiliations:** ^1^ Department of Behavioral Sciences and Social Medicine, College of Medicine, Florida State University, Tallahassee, Florida, USA.; ^2^ Department of Statistics, Florida State University, Tallahassee, Florida, USA.

**Keywords:** breast cancer, cancer stage, rural-urban disparities, medically underserved areas

## Abstract

***Purpose:*** To analyze the extent to which rural-urban differences in breast cancer stage at diagnosis are explained by factors including age, race, tumor grade, receptor status, and insurance status.

***Methods:*** Using the National Cancer Institute's Surveillance, Epidemiology, and End Results (SEER) 18 database, analysis was performed using data from women aged 50–74 diagnosed with breast cancer between the years 2013 and 2016. Patient rurality of residence was coded according to SEER's Rural-Urban Continuum Code 2013: Large Urban (RUCC 1), Small Urban (RUCC 2,3), and Rural (RUCC 4,5,6,7,8,9). Stage at diagnosis was coded according to SEER's Combined Summary Stage 2000 (2004+) criteria: Localized (0,1), Regional (2,3,4,5), and Distant (7). Descriptive statistics were analyzed, and variations were tested for across rural-urban categories using Kruskall–Wallis and Kendall's tau-b tests. Additionally, odds ratios (ORs) and 95% confidence intervals for the three ordinal levels of rural-urban residence were calculated while adjusting for other independent variables using ordinal logistic regression.

***Results:*** The rural residence category showed the largest proportion of women diagnosed with distant stage breast cancer. Additionally, we determined that patients with residence in both large and small urban areas had statistically significantly lower odds of higher stage diagnosis compared to rural patients even after controlling for age, race, tumor grade, receptor status, and insurance status.

***Conclusions:*** Rural women with breast cancer show small but statistically significant disparities in stage-at-diagnosis. Further research is needed to understand local area variation in these disparities across a wide range of rural communities, and to identify the most effective interventions to eliminate these disparities.

## Introduction

Every year more women are diagnosed with breast cancer than any other cancer excluding those of the skin.^1^ In 2020, it is estimated that 42,170 women in the United States will die from the disease.^1^ The United States has seen great reductions in breast cancer mortality, reflecting earlier detection through screening, patient education, and improved treatment.^2,3^ The survival rate for breast cancer is largely dependent on stage at diagnosis, with later stage breast cancer correlating to a poorer prognosis.^2,4,5^ The American Cancer Society reports that patients with localized or regional breast cancer have 5-year survival rates of 99% and 86% respectively, while patients who are diagnosed with distant breast cancer have a 5-year survival rate of only 27%.^6^

Although overall advances have been made in breast cancer outcomes in recent years, these advances have not accrued equally to all segments of the population. Sub-groups of patients differentiated by race, socioeconomic status, ethnicity, and geographic location continue to experience higher mortality rates of breast cancer.^2,7–11^ Specifically, rural communities face deeply rooted challenges not only due to their geographic location, but also due to racial-ethnic, economic, and health care system factors.^8,12,13^ Rural populations are often older, face higher levels of poverty, have lower levels of educational achievement and lack access to insurance and health care resources.^12,14,15^ Because of these factors, individuals in rural communities are vulnerable to higher levels of cancer mortality.^16^

With the survival rate for breast cancer highly dependent on stage at diagnosis, early detection is critical. Early detection with screening mammography allows earlier diagnosis and an overall mortality benefit.^3,17^ Rural women suffer significant disadvantages in obtaining these necessary mammography services compared to urban women.^8,16–18^

There can also be delays in diagnosis related to access issues in obtaining a biopsy after an abnormal mammogram. These disadvantages include but are not limited to decreased access to resources and increased distance to health care providers.^17,19,20^ Improving access to breast cancer screening and diagnostic resources among rural women could potentially decrease the number of rural patients presenting with later stage disease and improve overall mortality.

Therefore, we undertook this study to analyze the extent to which rural-urban differences in stage at diagnosis are explained by factors such as age, race, tumor grade, receptor status, and insurance status. Better understanding of what drives rural-urban differences in stage at diagnosis can guide future interventions designed to enhance breast cancer outcomes and achieve health equity for all women across the rural-urban continuum.

## Materials and Methods

### Database and study population

This study was conducted with the approval of the Florida State University Institutional Review Board (IRB) for Human Subjects Research. We obtained data on women diagnosed with breast cancer between the ages of 50 and 74 years through the National Cancer Institute's Surveillance, Epidemiology, and End Results (SEER) 18 database.^22^ The data were extracted using SEER*Stat software version (8.3.6.1).^52^ The SEER 18 database is a compilation of various population-based cancer registries that represent ∼28% of the U.S. population.^23^ The registries of the SEER program collect comprehensive data on patient demographics, primary tumor site, tumor morphology, stage at diagnosis, first course of treatment, and follow-up for vital status.^24^

Female patients with a breast cancer diagnosis were selected according to the Site and Morphology Site recode ICD-O-3/WHO 2008 provided by SEER. This selection include sites C500-C509 contained under the site group “Breast.”^25^ Patient age at diagnosis was defined according to SEER's age recode with single ages and 85+ variable.^26^ The study cohort for this cross-sectional analysis consisted of women between the ages of 50 and 74 years who were diagnosed with breast cancer between the years 2013 and 2016.

The study was restricted to this age range based on U.S. Preventive Services Task Force (USPSTF) guidelines for mammography screening, which recommends mammography for women age 50 to 74 years and individual decision making for screening at earlier ages. Our analyses do exclude younger and older women with breast cancer diagnoses, but we are interested in this age range for its relevance to mammography screening as an intervenable factor in rural communities. Approximately 49,511 women younger than age 50 and 48,993 women older than age 74 diagnosed with breast cancer between the years 2013 and 2016 were excluded from this study.

### Study variables

#### Outcome (dependent) variable

The primary outcome variable was breast cancer stage at diagnosis. This variable was coded according to the SEER Combined Summary Stage 2000 (2004+) criteria, explicitly according to site-specific guidelines for breast cancer.^27^ SEER's summary staging utilizes all relevant information available in a patient's medical record, making it a combination of the most precise clinical and pathological documentation of the extent of disease.^28^ The codes for this variable included the localized category, corresponding to SEER codes 0 and 1. SEER codes 2,3,4, and 5 were collapsed to create the regional category. Finally, SEER code 7 corresponded to the distant category. Patients with unknown, unspecified, or unstaged breast cancer were excluded from analysis.

#### Primary independent variable

The primary predictor (independent) variable of this study was rural versus urban residence of breast cancer patients. This variable was coded according to SEER's Rural-Urban Continuum Code (RUCC) 2013 criteria. These codes were originally developed by the United States Department of Agriculture (USDA) and schematically differentiate metropolitan (metro) counties by the population size of their metro area, and nonmetropolitan (nonmetro) counties by the degree of urbanization and adjacency to a metro area or areas.^29^

These codes reflect the rural or urban composition of the residential county for each patient at their time of diagnosis.^30^ This study utilized all nine RUCCs defined as follows: (1) counties in metro areas of 1 million population or more, (2) counties in metro areas of 250,000 population to 1 million population, (3) counties in metro areas of fewer than 250,000 population, (4) urban population of 20,000 or more, adjacent to a metro area, (5) urban population of 20,000 or more, not adjacent to a metro area, (6) urban population of 2,500 to 19,999, adjacent to a metro area, (7) urban population of 2,500 to 19,999, not adjacent to a metro area, (8) completely rural or less than 2,500 urban population, adjacent to a metro area and (9) completely rural or less than 2,500 urban population, not adjacent to a metro area.^29^

Our first category “Large Urban” was defined as counties in metro areas of ≥1 million population, which corresponded to RUCC 1. We collapsed RUCCs 2 and 3 into the second category defined as counties in metro areas of <1 million population or “Small Urban.” We then collapsed RUCCs 4,5,6,7,8, and 9 into the third category of counties in non-metro areas or “Rural.” Patients categorized under other, unknown or blank were excluded from analysis.

#### Other independent (control) variables

Patient race and origin were defined according to SEER's Race and Origin recode variable.^31^ This variable included categories for Non-Hispanic White, Non-Hispanic Black, and Hispanic. SEER's recodes for Non-Hispanic American Indian/Alaska Native, Non-Hispanic Asian or Pacific Islander, and Non-Hispanic Unknown Race (13,111 patients) were excluded from this analysis due to smaller cell sizes and statistically insignificant results for these race and origin groups.

Breast cancer grading was determined based on SEER's Grade recode. The grading and differentiation codes used in this study are defined in ICD-O-2; 1992.^32^ Categories included Grade I (well differentiated) and Grade II (moderately differentiated). SEER's categories for Grade III (poorly differentiated) and Grade IV (undifferentiated) were collapsed to form the category Grade III/IV (poorly differentiated/undifferentiated).

Patients with unknown grade were excluded from this study. Breast cancer receptor status was coded according to SEER's Breast Subtype (2010+) variable. Subtypes are defined by joint hormone receptor (HR; estrogen [ER] and progesterone [PR] receptor) and HER2 status.^33^ Categories under this variable included HR^+^/HER2^+^ (Luminal B), HR^−^/HER2^+^ (HER2 enriched), HR^+^/HER2^−^ (Luminal A), HR^−^/HER2^−^ (Triple Negative), and Unknown.

Patient's insurance status was coded according to SEER's Insurance Recode (2007+) variable derived from the national association of central cancer registries field, Primary Payer at Diagnosis.^34^ SEER's variable categories included the following: (1) Uninsured, (2) Any Medicaid (3) Insured, (4) Insured, No Specifics, and (5) Insurance Status Unknown. For the purpose of this study, we categorized as uninsured those originally coded as uninsured using the SEER variable criteria and those individuals under 65 years of age who were originally coded as insurance status unknown (as per NCI recommendations).^34^ Other values for this variable included those coded as having Medicaid, and a “Medicare, Private/Other Insurance” category that included SEER's categories for “Insured” and “Insured, No Specifics,” and individuals 65 years of age and older who were originally coded within SEER's insurance status unknown category.

### Statistical analysis

Descriptive statistics were first used to summarize the demographic characteristics for age at diagnosis, race and origin, tumor grade, receptor status, and insurance status by rural-urban residence. Variations in patient characteristics were tested across rural-urban categories using a Kendall's tau-b test when these characteristics were ordinal or categorial variables.^50^ A Kruskal–Wallis test was used when the characteristics were continuous variables.^50^

For our main analysis of the effect of each predictor on odds of being in a higher breast-cancer stage-at-diagnosis, we calculated the odds ratios (ORs) and 95% confidence intervals (CIs) for the three ordinal levels of rural-urban residency status while adjusting for other independent variables using ordinal logistic regression (OLR). Specifically, we calculated two ORs: the odds of stage = Local versus stage ≥ Regional (*i.e.*, Regional or Distant), and the odds of stage ≤ Regional (*i.e.*, Local or Regional) versus stage = Distant. For all statistical tests, a *p-*value <0.05 was considered as statistically significant.

All analyses were conducted utilizing R statistical software (version 3.6.1).^51^ We also performed additional analysis with interaction terms and analyses evaluating for multicollinearity.

## Results

Table 1 shows the distribution of clinical characteristics in patients at the time of diagnosis by rurality of residence. Rural women had the largest proportion of women diagnosed with distant breast cancer (4.94%) compared with women from small urban (4.36%) or large urban areas (4.24%). These differences were small, but statistically significant, and relevant on a population level. While most women were found to have moderately differentiated breast cancer, rural women had the largest proportion with poorly differentiated/undifferentiated breast cancer. For receptor status, proportions of women in both the large urban and small urban categories were more likely to have the treatment-responsive Luminal A (HR^+^/HER2^−^) cancers than rural women.

**Table 1.** Characteristics of Breast Cancer Patients by Rural/Urban Residence at Time of Diagnosis (*N* = 132,372)

Characteristic	Total *N* = 132,372 (%)	Large urban *n* = 81,233 (%)	Small urban *n* = 37,016 (%)	Rural *n* = 14,123 (%)	Test statistics	***p***
Age at diagnosis (years)^*^	132,372 (100)	81,233 (61)	37,016 (28)	14,123 (11)	0.023	<0.001
Tumor grade^*^					−0.007	0.005
Well-differentiated	33,036 (25)	19,755 (24)	9,674 (26)	3,607 (26)		
Moderately differentiated	59,975 (45)	37,338 (46)	16,535 (45)	6,102 (43)		
Poorly differentiated/undifferentiated	39,361 (30)	24,140 (30)	10,807 (29)	4,414 (31)		
Stage-at-diagnosis^a^					0.005	0.074
Local	91,783 (69)	56,392 (69)	25,758 (70)	9,633 (68)		
Regional	34,833 (26)	21,397 (26)	9,644 (26)	3,792 (27)		
Distant	5,756 (4.35)	3,444 (4.24)	1,614 (4.36)	698 (4.94)		
Receptor status					0.001	0.721
Luminal B	13,875 (11)	8,433 (11)	3,910 (11)	1,532 (11)		
HER2 enriched	5,748 (4)	3,460 (4)	1,628 (4)	660 (5)		
Luminal A	99,168 (75)	61,179 (75)	27,647 (75)	10,342 (73)		
Triple Negative	13,581 (10)	8,161 (10)	3,831 (10)	1,589 (11)		
Insurance status^*^					−0.025	<0.001
Medicaid	14,019 (11)	8,219 (10)	3,978 (11)	1,822 (13)		
Medicare or private/other insurance	115,308 (87)	71,247 (88)	32,154 (87)	11,907 (84)		
Uninsured	3,045 (2)	1,767 (2)	884 (2)	394 (3)		
Race^*^					−0.101	<0.001
White (1)	100,872 (76)	59,271 (73)	29,173 (79)	12,428 (88)		
Hispanic (3)	15,456 (12)	10,922 (13)	4,004 (11)	530 (4)		
Black (2)	16,044 (12)	11,040 (14)	3,839 (10)	1,165 (8)		

^*^ Statistically significant *p* < 0.05.

^a^ Dependent variable.

Larger proportions of rural women had both Triple Negative and HER2-enriched cancers compared to the urban categories. For insurance status, larger proportions of women in the rural category either had Medicaid or were uninsured compared to women in large or small urban areas. Most patients in all residence categories of rurality were white, with the rural category having the lowest proportions of Blacks and Hispanics compared to the large urban and small urban categories.

For OLR analysis with multiple predictors in Table 2, patients with residence in a rural area had significantly higher odds of higher stage diagnosis compared to large urban residing patients (OR = 1.07; with 95% CI: 1.03–1.11), as did patients living in small urban areas (OR = 1.04; 95% CI: 1.01–1.07). These data were produced using common ORs for Local versus Regional or Distant, and Local or Regional versus Distant stage at diagnosis.

**Table 2.** Estimated Main Regression Effects on the OR of More Advanced Stage at Diagnosis (OR of Regional/Distant vs. Local and OR of Distant vs. Local/Regional)

Predictor	Estimated coefficient (95% CI)	Standard error	OR (95% CI)	***p***
Age^*^	−0.015 (−0.017 to −0.013)	0.001	0.985 (0.983 to 0.986)	<0.001
Tumor grade				
Well-differentiated^*^	−1.05 (−1.09 to −1.02)	0.018	0.35 (0.34 to 0.36)	<0.001
Moderately differentiated^*^	−0.37 (−0.40 to −0.35)	0.015	0.69 (0.67 to 0.71)	<0.001
Poorly differentiated	REF		1.00	
Rural and urban				
Rural areas^*^	0.07 (0.03 to 0.11)	0.021	1.07 (1.03 to 1.11)	0.001
metro areas <1 million pop (small urban)^*^	0.04 (0.01 to 0.07)	0.015	1.04 (1.01 to 1.07)	0.008
Metro areas >1 million pop (large urban)	REF		1.00	
Receptor status				
Luminal B^*^	−0.09 (−0.15 to −0.02)	0.032	0.92 (0.86 to 0.98)	<0.001
Luminal A^*^	−0.16 (−0.22 to −0.11)	0.029	0.85 (0.80 to 0.90)	<0.001
Triple Negative^*^	−0.30 (−0.37 to −0.24)	0.032	0.74 (0.69 to 0.79)	<0.001
HER2 enriched (HR^−^/HER2^+^)	REF		1.00	
Insurance status				
Medicaid	0.04 (−0.04 to 0.12)	0.041	1.04 (0.96 to 1.13)	0.337
Medicare or private/other insurance^*^	−0.34 (−0.42 to −0.27)	0.038	0.71 (0.66 to 0.76)	<0.001
Uninsured	REF		1.00	
Race				
Non-Hispanic Black^*^	0.23 (0.18 to 0.27)	0.022	1.26 (1.20 to 1.31)	<0.001
Hispanic^*^	0.14 (0.10 to 0.19)	0.022	1.16 (1.11 to 1.21)	<0.001
Non-Hispanic White	REF		1.00	

^*^ *p* < 0.05.

CI, confidence interval; HR, hormone receptor; OR, odds ratio.

Other covariates had a more dramatic impact on stage-at diagnosis. For example, patients with well-differentiated tumors had 65% lower odds of being diagnosed at regional or distant stage (OR = 0.35; 95% CI: 0.34–0.36) and patients with moderately differentiated tumors had 31% lower odds of being diagnosed at distant stage (OR = 0.69; 95% CI: 0.67–0.71) relative to those with poorly differentiated tumors. Certain categories of HR status were also associated with much lower ORs for later-stage diagnosis. Since the correlations among large urban and small urban with other predictors are close to zero (Table 3), no evidence of multicollinearity among independent variables was detected.

**Table 3.** Correlations Among the Pairs of Large Urban, Small Urban, and Other Predictors

	Large urban	Small urban
Age	0.03	0.01
Well-differentiated	0.00	0.02
Moderately differentiated	−0.01	−0.01
Luminal B	0.00	0.00
Luminal A	−0.01	0.00
Triple Negative	0.01	0.00
Medicaid	0.03	0.00
Other insurance	−0.03	0.00
Non-Hispanic Black	−0.04	−0.03
Hispanic	−0.09	−0.02

Relative to uninsured patients, patients with Medicare or Private/Other insurance had 29% lower odds of later stage-at-diagnosis (OR = 0.71; 95% CI: 0.66–0.76), but Medicaid patients had no statistical differences in OR for later stage-at-diagnosis (OR = 1.04; 95% CI: 0.96–1.13). Race-ethnicity was also a significant predictor of stage at diagnosis in the OLR model, with Hispanic women (OR = 1.16; 95% CI: 1.11–1.21) and especially Black women (OR = 1.26; 95% CI: 1.20–1.31) having statistically significant higher odds of having later-stage diagnosis relative to white women.

In Table 4, two interaction terms, Small Urban: Non-Hispanic Black and Small and Urban: Hispanic are statistically significant compared to Large Urban: Non-Hispanic White. Thus, we found heterogeneities among races in large urban and small urban areas. Also, rural areas as a main effect showed greater impact on higher stage-at-diagnosis patients compared to patients from large urban areas (Table 2). Thus, these strong main effects demonstrated that the interaction effects of races between large urban and rural areas were insignificant (Table 4).

**Table 4.** Interaction Effects

Predictor	Estimated coefficient (95% CI)	Standard error	OR (95% CI)	***p***
Small urban: Non-Hispanic Black^*^	−0.09 (−0.17 to −0.01)	0.04	0.91 (0.84 to 0.99)	0.030
Rural: Non-Hispanic Black	0.02 (−0.11 to 0.15)	0.06	1.02 (0.89 to 1.16)	0.763
Small urban: Hispanic^*^	−0.12 (−0.21 to −0.04)	0.04	0.88 (0.81 to 0.96)	0.026
Rural: Hispanic	0.01 (−0.18 to 0.18)	0.09	1.00 (0.83 to 1.20)	0.966

^*^ *p* < 0.05.

## Discussion

This study confirms a small but statistically significant rural-urban disparity in stage at diagnosis of breast cancer using the national, multi-registry SEER database. It also confirms a continuum of disparity from rural to small urban to large urban areas and quantifies the strength of this association even after controlling for other known factors such as age, race, insurance status, tumor grade, and tumor receptor status. Our findings highlighted that residence in a rural area corresponded to an increased likelihood of being diagnosed with a later stage breast cancer compared to residence in a large or small urban area.

The mechanism by which rural-urban disparities in stage at diagnosis may occur include, but are not limited to, disparities in access to and utilization of mammography screening, and potential delays between abnormal screening test and biopsy-proven diagnosis. Our finding of a rural disparity in tumor grade (*i.e.*, rural women more likely to have poorly differentiated tumors) also suggests the potential effects of either lifestyle or environmental factors on the aggressiveness of the cancer itself and its impact on stage at diagnosis.

Rural populations are not at increased risk for developing breast cancer. Evidence suggests that as urbanicity increases, so does the incidence of breast cancer.^11,13,35–37^ However, for women in many rural populations, the breast cancer mortality rate is not following the national trend of decreasing mortality, but rather is stagnant or even increasing.^36^ Previous studies have shown varying results with regard to the relationship between breast cancer stage at diagnosis and rural-urban residence. Several studies have concluded that rural women are diagnosed more often with later stage breast cancer.^38–40^

However, this may not apply to all sub-groups of rural women.^41^ For example, in a recent study on postmenopausal women utilizing data from the Women's Health Initiative, researchers concluded that rural-urban residence was not significantly associated with breast cancer stage at diagnosis.^15^ There may also be state-level variation. Breast cancer data from the Illinois State Cancer Registry suggested an “urban disadvantage” in which later-stage presentation is more likely in urban than rural women.^42^ Overall however, a systematic review including 24 studies and meta-analysis including 21 studies (*N* = 879,660) performed using peer reviewed literature on rural and urban differences in staging at diagnosis of breast cancer, concluded that rural women were more likely than urban women to be diagnosed with late-stage breast cancer.^39^

The disparities in breast cancer stage-at-diagnosis are complicated and may be explained by multiple factors. However, to improve breast cancer outcomes for women across the country, especially rural women, increasing the access to and use of screening mammography may be an important area of focus. Overall, rates of screening mammography have increased for women across the United States. However, previous studies suggest that rural women in the United States are less likely than urban women to have regular screening mammograms.^21,43–45^

Not all rural communities are the same, however, and therefore the relationship between rurality and breast cancer will be driven differently depending on a multitude of factors. For example, in the Appalachia region, lower breast cancer screening rates in areas considered “highly deprived” were associated with later stage breast cancer diagnosis.^21^ Racial-ethnic diversity, insurance rates, and population density also vary dramatically across rural areas in different regions and local areas across the United States.

The American Cancer Society and other studies have concluded that mammographic screening can help to detect breast cancer in its earlier stages, which saves lives.^3,46^ Unfortunately, research shows that rural women face challenges with scarcity of resources and physicians and long distances to medical facilities when it comes to screening mammography.^13,45,47^

This disparity highlights the need for developing solutions to the many barriers rural women face in access to mammography (and appropriate diagnostic follow-up) among other health care barriers, which could result in better care, earlier breast cancer diagnosis, and improvement in treatment outcomes. For example, recent studies on mobile mammography clinics show that these programs benefit underserved women, many of which live in rural areas, and may serve as a path toward eliminating breast cancer disparities by improving access to screening to detect breast cancer earlier when it is more likely to be curable.^44,48,49^

### Limitations

Limitations of this study are tied to the known limitations of using cancer registry data, without access to the individual's medical record. We were not, for example, able to assess the individual's history or timing of undergoing screening mammography, or the time between mammography and diagnosis. The absolute rural-urban differences were small, compared with differences in stage at diagnosis by tumor grade or other covariates, but were statistically significant and may represent an intervenable risk for decreased survival at a population level.

There is tremendous heterogeneity in rural areas ranging from the Appalachian region to the Deep South to upstate New York or to vast frontier communities of the West. Additionally, it is important to note that SEER data may not be nationally representative, limiting the generalizability of these study findings. Further research will be needed to tease out the specific elements of rurality such as health care system capacity, insurance status, population density, and distance to screening or biopsy services, which drive differences in stage at diagnosis.

## Conclusion

In conclusion, rural women do appear to suffer disparities in stage at diagnosis, even after controlling for known biological factors such as age, race, ethnicity, insurance status, tumor grade, and receptor status. Further research is needed not only to identify the mechanisms by which this occurs and the variation in these disparities across heterogenous rural communities, but also the community-specific interventions by which these rural urban disparities might be eliminated.

## Abbreviations Used

CIs: confidence intervals
ER: estrogen receptor
HR: hormone receptor
IRB: Institutional Review Board
OLR: ordinal logistic regression
ORs: odds ratios
PR: progesterone receptor
RUCC: Rural-Urban Continuum Code
SEER: Surveillance, Epidemiology, and End Results
USDA: United States Department of Agriculture
USPSTF: U.S. Preventive Services Task Force
